# circRNA_0000285 knockdown suppresses viability and promotes apoptosis of cervical cancer cells by sponging microRNA-654-3p

**DOI:** 10.1080/21655979.2022.2037870

**Published:** 2022-02-15

**Authors:** Sisi Zhang, Yingping Xu, Qingyu Zheng

**Affiliations:** a Department of Obstetrics and Gynaecology, Jingzhou Hospital, Yangtze University, Jingzhou, Hubei, P.R. China; b Department of Obstetrics and Gynecology, Renmin Hospital, Hubei University of Medicine, Shiyan, P.R, China; c Department of Ultrasound, Zhijiang People’s Hospital, Zhijiang, P.R, China

**Keywords:** Cervical cancer, circular RNA_0000285, microRNA-654-3p

## Abstract

Cervical cancer (CC) is one of the most common gynecological tumors worldwide. Several studies have reported that circular RNAs (circRNAs) play important roles in various types of diseases, including cancer. Thus, the present study aimed to investigate the role of circRNA_0000285 in CC development. Dual-luciferase reporter and RNA pull-down assays were performed to verify the binding region between circRNA_0000285 and miR-654-3p. The expression levels of circRNA_0000285 and miR-654-3p were analyzed in CC and the corresponding normal tissues, as well as in SiHa, HeLa, and NC104 cells using reverse transcription-quantitative polymerase chain reaction (RT-qPCR). In addition, the effect of circRNA_0000285 inhibition on cell viability, apoptosis, and the expression of apoptosis-related markers was assessed using MTT (3-(4,5-dimethylthiazol-2-yl)-2,5-diphenyltetrazolium bromide), flow cytometry, and Western blotting assays, respectively. The results verified that miR-654-3p directly targeted circRNA_0000285 expression. circRNA_0000285 was overexpressed and miR-654-3p expression was downregulated in CC tissues and cells compared to that in control. Moreover, circRNA_0000285 knockdown suppressed the viability and promoted the apoptosis of CC cells, which was accompanied by the downregulated and upregulated expressions B-cell lymphoma-2 (*Bcl-2*) and Bcl-2 associated X (*Bax*), respectively. The ratio of Bax/Bcl-2 levels also increased following circRNA_0000285 knockdown. However, these findings were abrogated after miR-654-3p inhibitor treatment. Hence, circRNA_0000285 knockdown suppressed cell viability and promoted apoptosis by targeting miR-654-3p in CC.

## Graphical abstract

**Figure:**
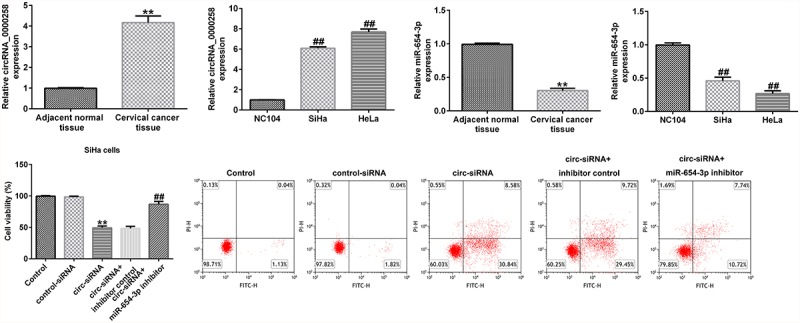

## Introduction

Cervical cancer (CC) is one of the most frequent gynecological tumors worldwide, with ~500,000 newly diagnosed patients reported every year [1,2]. The incidence of CC continues to increase annually [3]. Human papillomavirus (HPV) infection is suspected to be the primary cause of CC [4]. Even though vaccination and early treatment procedures can reduce CC cases, the prognosis and survival rate of patients with advanced CC remains dismal [5]. Thus, it is of great significance to enhance the current understanding of CC development to identify the novel biomarkers and therapeutic targets for patients.

Circular RNAs (circRNAs), which form a covalently closed loop in contrast to linear RNAs, have attracted significant interest within the field of non-coding RNA research [6,7]. Numerous reports have shown that circRNAs play significant roles in various types of diseases, including cancer, diabetes, and cardiovascular diseases [8–10]. Furthermore, circRNAs were found to be abnormally expressed in numerous types of tumor cells, suggesting their potential as targets for cancer treatment [11]. circRNAs modulate multiple biological processes in cancer cells, including proliferation, survival, and invasion [12–15]. In addition, circRNA_0000285 has been suggested to play a carcinogenic role in many types of cancer [16–20]. In addition, the downregulation of circRNA_0000285 expression is an important prognostic biomarker for bladder cancer [17]. circRNA_0000285 also promotes laryngeal cancer progression by activating the Wnt signaling pathway [18]. Zhang et al. reported that circRNA_0000285 promotes osteosarcoma progression by sponging miRNA-599 [19]. Moreover, Long et al. indicated that circRNA_0000285 regulates proliferation, migration, invasion and apoptosis of osteosarcoma by miR-409-3p/insulin-like growth factor-binding protein 3 (IGFBP3) axis [20]. Nevertheless, to the best of our knowledge, the effects of circRNA_0000285 in CC and its potential molecular mechanisms remain to be determined.

miRNAs are noncoding RNAs that regulate target gene expression by binding to the 3ʹ-UTR, resulting in mRNA degradation or translational repression [21]. Multiple studies have suggested that miRNAs may play important roles in numerous types of human cancer, including CC [22–24]. Abnormal expression of miRNAs affects the expression of both cancer-promoting and cancer-suppressing genes, thereby altering cell viability, invasion, and migration potential of the CC cells [25]. In addition, accumulating evidence has shown that circRNAs may interact with miRNAs or RNA-binding proteins to regulate numerous biological functions, such as cell proliferation, apoptosis, and metastasis [26]. A previous study reported that circRNA_0000285 promotes osteosarcoma progression by sponging miR-599 [19]. Furthermore, circRNA_101996 acted as a sponge for miR‐8075, which relieved the repressive effect of miR‐8075 on Xenopus kinesin-like protein 2 (*TPX2*) expression, subsequently upregulating *TPX2* expression and promoting tumor progression [27]. Furthermore, circRNA_0000285 induced damage to podocytes by targeting miR-654-3p and regulating mitogen-activated protein kinase 6 (*MAPK6*) in diabetic nephropathy [28]. Nevertheless, the effects and underlying mechanisms of circRNA_0000285 and miR-654-3p on CC progression are largely unknown and require further investigation.

In the present study, we hypothesized that circRNA_0000285 exists as an oncogene in CC by regulating the expression of miR-654-3p. Therefore, we designed this study to illustrate the physiological function and mechanism of circRNA_0000285 in CC development.

## Materials and methods

### Patient information and sample collection

CC and the adjacent normal tissues were obtained from 30 patients at the Jingzhou Central Hospital (Jingzhou, China). Tissues were stored in liquid nitrogen after surgical removal until required for further analysis. The experimental protocol was approved by the Ethics Committee of Jingzhou Central Hospital. Written consent was obtained from the patient prior to the operation.

### Cultivation of cell lines

The CC cell lines, SiHa and HeLa, and the normal cervical epithelium cell line, NC104, were purchased from the American Type Culture Collection (ATCC). All cells were cultured in Dulbecco’s Modified Eagle Medium (DMEM; Thermo Fisher Scientific, MA, USA) containing 10% fetal bovine serum (FBS; Thermo Fisher Scientific, MA, USA) and 1% penicillin-streptomycin (Merck KGaA, Darmstadt, Germany), and maintained at 37°C in a humidified 5% CO_2_ incubator.

### Cell transfection

To silence circRNA_0000258 expression in CC cells, interference technology was performed [29]. The small interfering RNA (siRNA) used as control (control-siRNA; 5ʹ-AAGACAUUGUGUGUCCGCCTT-3ʹ), circRNA_0000258-siRNA (circ-siRNA; 5ʹ-CCCCAGCUAUUCAAGUGUAAA-3ʹ), inhibitor control (5ʹ-AAGUCAGGUGAUGGACAGCAUA-3ʹ), and miR-654-3p inhibitor (5ʹ-AAGGUGAUGGUCAGCAGACAUA-3ʹ) were synthesized by Shanghai GenePharma Co., Ltd., Shanghai, China. SiHa/HeLa cells were transfected with each oligonucleotide using Lipofectamine® 2000 transfection reagent (Thermo Fisher Scientific, MA, USA), by following the manufacturer’s instructions.

### Dual-luciferase reporter assay *[30]*

A previous study predicted a binding site between circRNA_0000258 and miR-654-3p [28]. To verify target binding, pGL3 luciferase reporter vectors with the wild-type (WT) or mutant (MUT) sequence of circRNA_0000285 were established. 293 T cells were seeded into 24-well plates and transfected with vectors harboring WT or MUT luciferase reporters, along with miR-654-3p mimic or mimic control using Lipofectamine® 2000. After 48 h of transfection, Dual-Luciferase® Reporter Assay system (Promega, WI, USA) was used to assess the relative luciferase activity.

### RNA pull-down assay *[31]*

Specific circRNA_0000285 and control probes were obtained from Shanghai GenePharma Co., Ltd., Shanghai, China. Cells were lysed and then incubated with the specific circRNA_0000285 or negative control probes at 4°C overnight. After incubation, the cells were washed with RNA immunoprecipitation (RIP) Wash Buffer, and an RNeasy Mini kit (QIAGEN, Hilden, Germany) was used to quantify the amount of RNA complex bound to the beads. Reverse transcriptase-quantitative PCR (RT-qPCR) was subsequently used to assess the levels of miR-654-3p and circRNA_0000285.

### RT-qPCR

Total RNA was extracted from tissue samples and SiHa, HeLa, and NC104 cells using TRIzol® reagent (Thermo Fisher Scientific, MA, USA). Total RNA (2 µg) was reverse transcribed into cDNA using HiScript™ II Q RT SuperMix (Vazyme Biotech Co., Ltd., China). qPCR was carried out using ChamQ Universal SYBR qPCR Master mix (Vazyme Biotech Co., Ltd., China) on an ABI 7900HT Fast Real-Time PCR System with 384-Well Block Module (Thermo Fisher Scientific, MA, USA). The following thermal cycling conditions were used for qPCR: initial denaturation for 5 min at 95°C, followed by 38 cycles of denaturation for 5 s at 95°C; amplification for 10s at 60°C; and a final extension for 10 min at 72°C. Relative circRNA/miRNA expression levels were quantified using the 2^−ΔΔCq^ method [32] and normalized to GAPDH/U6, the endogenous control. The primer sequences used for qPCR were as follows:

circRNA_0000285 forward, 5ʹ-TACCTCTGCAGGCAGGAACT-3 ʹ;

reverse, 5ʹ-TCACATGAATTTAGGTGGGACTT-3ʹ;

miR-654-3p forward, 5ʹ-GGGATGTCTGCTGACCA-3ʹ;

reverse, 5ʹ-CAGTGCGTGTCGTGGA-3ʹ;

U6 forward, 5ʹ-CTCGCTTCGGCAGCACA-3ʹ;

reverse, 5ʹ-AACGCTTCACGAATTTGCGT-3ʹ;

GAPDH forward, 5ʹ-TCAACGACCACTTTGTCAAGCTCA-3ʹ;

reverse, 5ʹ-GCTGGTGGTCCAGGGGTCTTACT-3ʹ.

### Western blotting *[33]*

Total protein was extracted using RIPA lysis buffer (Beyotime, Shanghai, China) and quantified using a BCA protein assay kit (Beyotime, Shanghai, China). Subsequently, the total protein was resolved using 8% SDS-PAGE and transferred to a PVDF membrane. After blocking with 5% skim milk in PBST (1x Phosphate-buffered saline with 0.1% Tween® 20 detergent) for 1 h, the membranes were incubated with anti-Bax, anti-Bcl-2, and anti-GAPDH primary antibodies (1:1000; Abcam, Cambridge, UK) overnight at 4°C. After washing with TBST (1X Tris-buffered saline with 0.1% Tween® 20 detergent), the membranes were incubated with secondary antibodies for 1 h. Protein bands were visualized, and their density was assessed using ImageJ software version 1.46.

### MTT assay *[34]*

Cells were seeded into 96-well plates at a density of 1 × 104 cells/well and cultured for 24 h prior to transfection. After this treatment, 10 µL MTT solution was added to the medium and the cells were incubated for 4 h. The supernatant was subsequently discarded, and the cells were treated with 100 μL dimethyl sulfoxide (DMSO). The plates were thereafter incubated for 10 min with mild oscillation to dissolve the formazan crystals. The absorbance was recorded as OD_570 nm_ using a VersaMax ELISA microplate reader (Molecular Devices, LLC, CA, USA).

### Flow cytometric analysis of apoptosis *[35]*

The Annexin V-FITC/PI apoptosis detection kit (Beyotime Biotechnology, Shanghai, China) was used to analyze cell apoptosis. After transfection for 48 h, cells were cultivated with 10 μL Annexin V-FITC and 5 μL PI in dark for 15 min. Apoptotic cells were assessed using a FACSCalibur flow cytometer (BD Biosciences, NJ, USA).

### Statistical analysis

SPSS software (version 22.0) was used for statistical analysis. The statistical significance of the difference between groups was determined using Student’s t-test or one-way analysis of variance (ANOVA) followed by Tukey’s post hoc test. Data are presented as mean ± standard deviation (SD) from three independent experiments. Statistical significance was set at p < 0.05.

## Results

### circRNA_0000285 acts as a sponge for miR-654-3p

**Figure 1.**
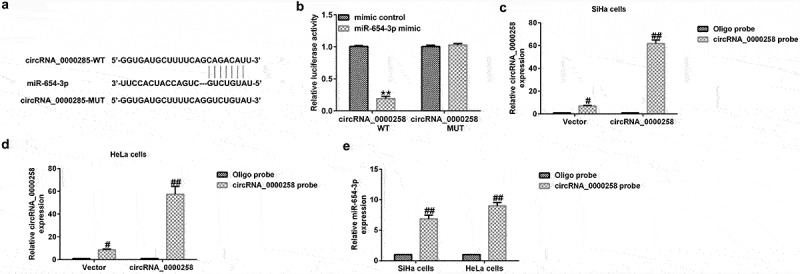
**circRNA_0000285 serves as a sponge for miR-654-3p**. (a) Predicted circRNA_0000285 binding site within the miR-654-3p WT or MUT 3ʹ-UTR. (b) Relative luciferase activity detected following the co-transfection of the luciferase reporter construct containing the miR-654-3p 3ʹ-UTR sequence with the WT or MUT circRNA_0000285 binding site. (c-e) circRNA_0000285-specific probe used to pull-down circRNA_0000285 and enrich miR-654-3p. P < 0.05, **P < 0.01 vs. control.

circRNAs have been shown to serve as competing endogenous RNAs for miRNAs during tumor progression [13,15]. The relationship between circRNA_0000285 and miR-654-3p is shown in Figure 1a. In the present study, cells were exposed to WT or MUT luciferase reporter-carrying vectors miR-654-3p mimics or mimic control. As shown in Figure 1b, the miR-654-3p mimic significantly reduced WT circRNA_0000285 luciferase activity, but had no effect on the activity of MUT circRNA_0000285. To further verify the binding, a circRNA_0000285-specific probe was used, which was able to pull-down circRNA_0000285 (Figure 1c and d). Furthermore, miR-654-3p levels were upregulated by the circRNA_0000285-specific probe (Figure 1e). These results suggest that miR-654-3p directly interacts with circRNA_0000285.

*circRNA_0000258 expression levels were upregulated, whereas miR-654-3p levels were downregulated in CC tissues and cells*

**Figure 2.**
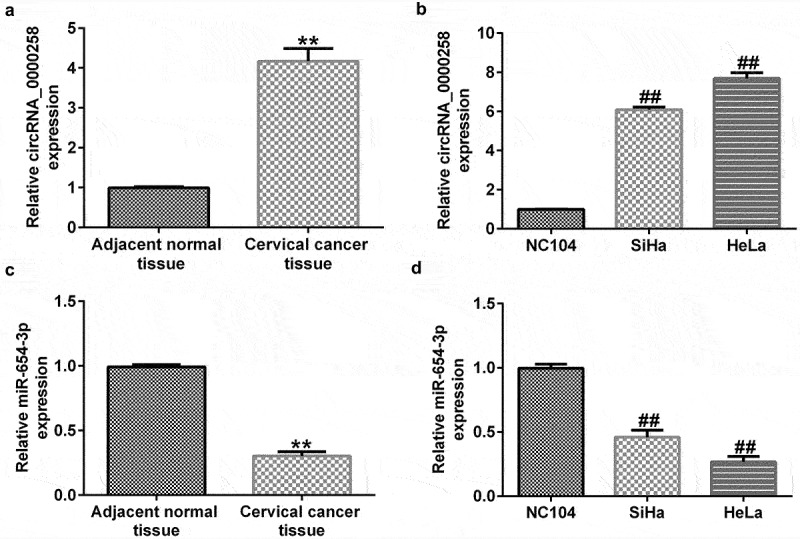
**circRNA_0000258 expression levels are upregulated, while miR-654-3p are down-regulated in CC tissue and cells**. (a) Levels of circRNA_0000258 in 30 CC and adjacent normal tissue samples assessed via RT-qPCR. (b) RT-qPCR analysis of circRNA_0000258 in SiHa, HeLa and NC104 cells. (c) Levels of miR-654-3p in 30 CC and adjacent normal tissues evaluated using RT-qPCR. (d) RT-qPCR analysis of miR-654-3p in SiHa, HeLa and NC104 cells analyzed using RT-qPCR. P < 0.05, **P < 0.01 vs. control.

To illustrate the roles of circRNA_0000285 and miR-654-3p in CC, RT-qPCR was used to evaluate their expression levels in CC tissues and cell lines. As shown in Figure 2a, the levels of circRNA_0000258 were significantly higher in CC tissues than in normal tissues. Similarly, the expression levels of circRNA_0000258 were markedly upregulated in SiHa and HeLa cells compared to that in NC104 cells (Figure 2b). Conversely, miR-654-3p was relatively downregulated in CC tissues compared to that in normal tissues (Figure 2c). Consistent with these findings, the levels of miR-654-3p were downregulated in SiHa and HeLa cells compared to NC104 cells (Figure 2d).

### circRNA_0000258 negatively regulates the levels of miR-654-3p in SiHa and HeLa cells

**Figure 3.**
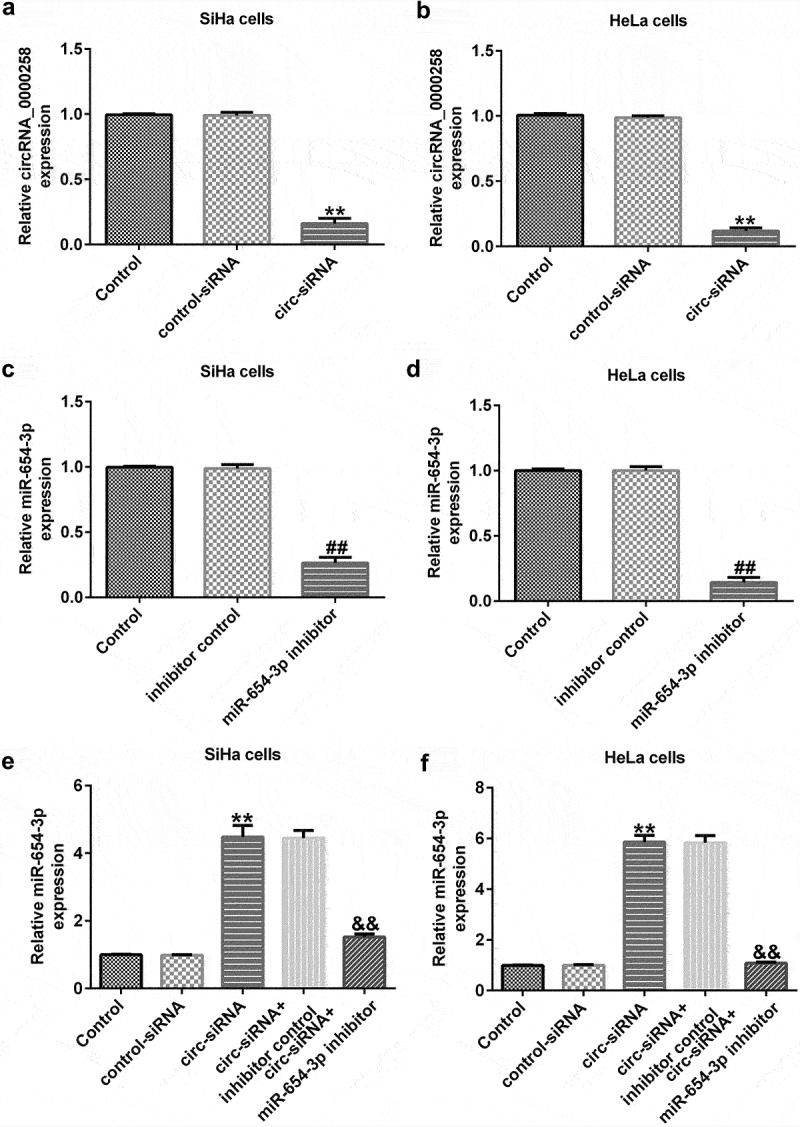
**circRNA_0000258 regulates miR-654-3p levels in SiHa and HeLa cells**. Expression levels of circRNA_0000258 analyzed in the circ-siRNA and control-siRNA groups in SiHa (a) and HeLa cells (b). Levels of miR-654-3p measured in the miR-654-3p inhibitor and inhibitor control groups in SiHa (c) and HeLa cells (d). (e, f) Levels of miR-654-3p in different groups. P < 0.05, **P < 0.01 vs. control.

To analyze the correlation between circRNA_0000258 and miR-654-3p in CC, the control siRNA, circ-siRNA, inhibitor control, or miR-654-3p inhibitor were transfected into SiHa and HeLa cells. After transfection, the level of circRNA_0000258 was lower in circ-siRNA-transfected cells than in the cells of the control siRNA group (Figure 3a and b). Moreover, the miR-654-3p inhibitor markedly downregulated miR-654-3p in SiHa and HeLa cells (Figure 3c and d). Notably, miR-654-3p was upregulated after circ-siRNA transfection, but this increase was repealed by the miR-654-3p inhibitor (Figure 3e and f). These data indicate that circRNA_0000258 may negatively regulate the levels of miR-654-3p in SiHa and HeLa cells.

*Knockdown of circRNA_0000258 inhibits cell growth and promotes cell apoptosis by upregulating miR-654-3p in CC cells*

**Figure 4.**
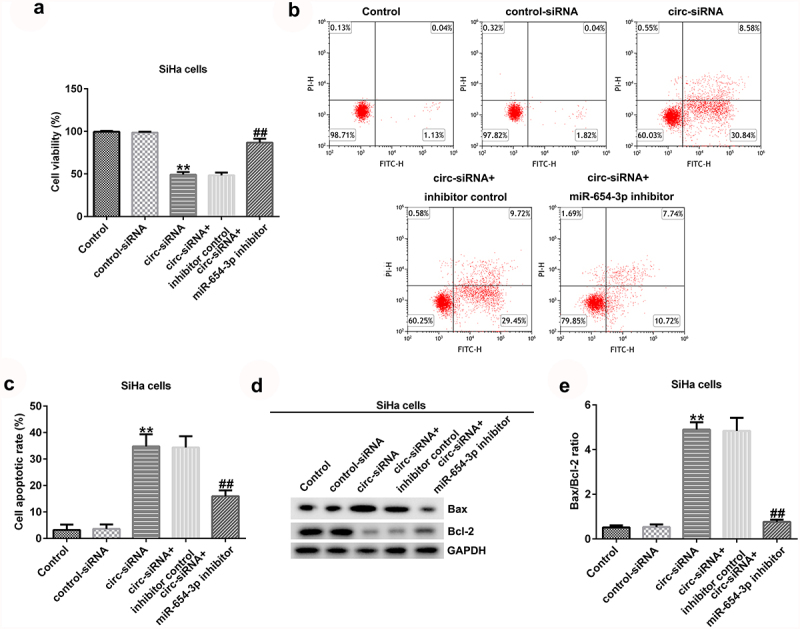
**Knockdown of circRNA_0000258 regulates SiHa cell viability and apoptosis via upregulation of miR-654-3p expression**. SiHa cells were transfected with control-siRNA, circ-siRNA, circ-siRNA + inhibitor control or circ-siRNA + miR-654-3p inhibitor. (a) Effect of circ-siRNA transfection for 48 h on SiHa cell viability evaluated by MTT assay. (b, c) Effect of circ-siRNA on SiHa cell apoptosis analyzed using flow cytometry. (d) Western blotting to detect Bax and Bcl-2 protein expression. (e) Ratio of Bax/Bcl-2. P < 0.05, **P < 0.01 vs. control.

**Figure 5.**
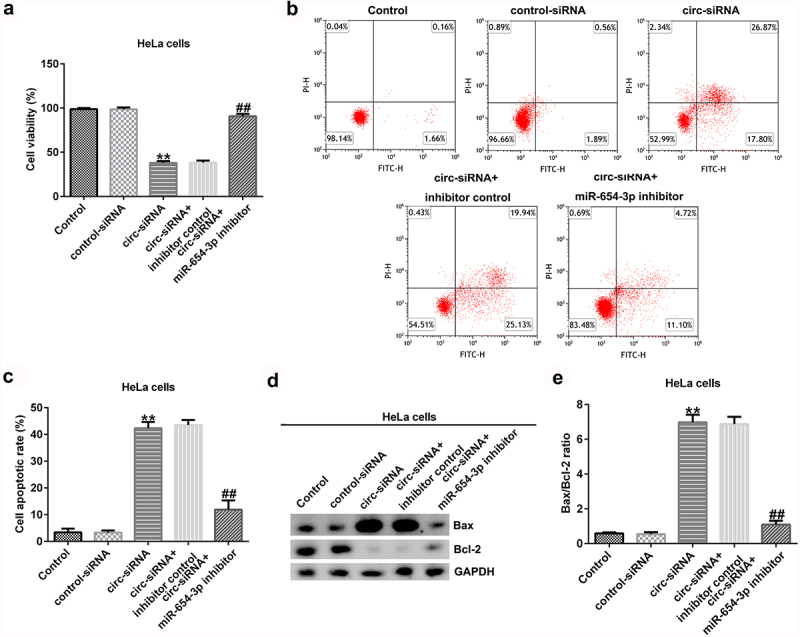
**Knockdown of circRNA_0000258 regulates HeLa cell viability and apoptosis via upregulation of miR-654-3p expression**. HeLa cells were transfected with control-siRNA, circ-siRNA, inhibitor control or miR-654-3p inhibitor. (a) Cell viability assessed using MTT assay. (b, c) Flow cytometry analysis of apoptotic cells. (d) Expression of Bax and Bcl-2 detected using Western blotting. (e) Ratio of Bax/Bcl-2. P < 0.05, **P < 0.01 vs. control.

To further explain the effects of circRNA_0000258 and miR-654-3p on CC, the control siRNA, circ-siRNA, inhibitor control, or miR-654-3p inhibitor were transfected into SiHa and HeLa cells. MTT and flow cytometric analyses were subsequently used to determine the role of circRNA_0000258 in the viability and apoptosis of SiHa and HeLa cells after 48 h of transfection. The results indicated that circ-siRNA significantly decreased the viability of SiHa cells (Figure 4a) by promoting the apoptosis (Figure 4b and c). Furthermore, *Bax* was overexpressed and *Bcl-2* levels were low following the transfection of circ-siRNA into SiHa cells (Figure 4d). Moreover, circ-siRNA increased the Bax/Bcl-2 ratio in SiHa cells (Figure 4e). However, these observations were reversed after the transfection with miR-654-3p inhibitor. Similar results were obtained in HeLa cells (Figure 5), suggesting that circRNA_0000258 may influence cell viability and apoptosis by down-regulating miR-654-3p in CC.

## Discussion

CC is one of the most common gynecological tumors worldwide [1,2]. There is increasing evidence demonstrating the essential role of circRNAs in CC progression. For example, Xu et al. [36] suggested that hsa_circ_0031288 levels were overexpressed in CC tissues and cells, which decreased the viability, invasion, and migration of CC cells. In addition, hsa_circRNA_101996 increased the CC cell viability and invasion by altering *TPX2* expression [27] mediated by the inhibition of miR-8075 expression. Our findings validate the inhibitory role of circRNA_0000285 knockdown in CC. In addition, miR-654-3p was verified as a direct target of circRNA_0000285, and the knockdown of circRNA_0000285 was shown to suppress CC development.

circRNAs are a type of endogenous RNA that have been reported to mediate gene expression by sponging target miRNAs, affecting target gene expression [37]. For example, circRNA_0000285 was found to induce cell invasion by altering miR-181b expression in pancreatic cancer cells [38]. In this study, we identified a binding site between circRNA_0000285 and miR-654-3p, and further assays verified that miR-654-3p was a direct target of circRNA_0000285.

To explain the functions of circRNA_0000285 and miR-654-3p in CC, we first quantified circRNA_0000285 and miR-654-3p expression in CC tissues and cell lines using RT-qPCR. We found that circRNA_0000285 was upregulated and miR-654-3p was downregulated in CC tissues and cells compared to the respective controls. The present results are consistent with those of previous studies [16,39], which have also demonstrated that circRNA_0000285 levels were higher in CC tissues and cells than in control tissues. To the best of our knowledge, this is the first study to illustrate the relationship between circRNA_0000285 and miR-654-3p and their effects on CC.

We further explained the underlying mechanisms of circRNA_0000285 and miR-654-3p in the progression of CC, by transfecting the CC cells with control-siRNA, circ-siRNA, inhibitor control, or miR-654-3p inhibitor. The results of the RT-qPCR assay demonstrated that circ-siRNA notably downregulated circRNA_0000285 levels, while the expression of miR-654-3p was suppressed in CC cells. Furthermore, circRNA_0000285-siRNA enhanced the miR-654-3p expression in CC cells, but this effect was abolished after addition of miR-654-3p inhibitor. Previous research has demonstrated that circRNA_0000285 increased the cell proliferation and metastasis of non-small cell lung cancer cells by altering miR-144 expression [40]. Similarly, knockdown of circRNA_0000285 suppressed CC cell proliferation, migration, and invasion by downregulating *FUS* expression [16]. Moreover, another study indicated that inhibition of circRNA_0000285 blocked the CC progression by upregulating miR197-3p and suppressing *ELK1* [39]. Our results suggest that inhibition of circRNA_0000285 decreased the viability and promoted the apoptosis of CC cells, which was partially consistent with the findings of previous studies [16,39]. However, these observations were abrogated in the presence of miR-654-3p inhibitor. In conclusion, these data suggest that circRNA_0000285 may alter CC cell growth and apoptosis by targeting miR-654-3p, indicating a latent role of miR-654-3p in the function of circRNA_0000285 in CC. However, the present study was limited in terms of cell lines, and more cell lines need to be examined to further understand the role of circRNA_0000285 and miR-654-3p in CC. In addition, the potential target of miR-654-3p should be analyzed to clarify the relevance of circRNA_0000285 and miR-654-3p in CC. Also, the relationship between miR-654-3p and *FUS* or *ELK1* requires further investigation.

## Conclusion

The results of current study suggest that knockdown of circRNA_0000285 potentially suppresses CC cell viability and promotes cell apoptosis by sponging miR-654-3p, implying a latent role of the circRNA_0000285/miR-654-3p axis in CC progression. These findings provide novel insights in the pathogenesis of CC and suggest new targets for the treatment of CC.

## Data Availability

The datasets used and/or analyzed during the present study are available from the correspond

## Research Highlights

circRNA_0000285 acts as a sponge for miR-654-3p;

circRNA_0000258 expression levels were upregulated, whereas miR-654-3p levels were downregulated in CC tissues and cells;

Knockdown of circRNA_0000258 inhibits cell growth and promotes cell apoptosis by upregulating miR-654-3p in CC cells.
